# Effort and Fatigue-Related Functional Connectivity in Mild Traumatic Brain Injury

**DOI:** 10.3389/fneur.2018.01165

**Published:** 2019-01-18

**Authors:** Amy E. Ramage, David F. Tate, Anneliese B. New, Jeffrey D. Lewis, Donald A. Robin

**Affiliations:** ^1^Department of Communication Sciences and the Interdisciplinary Program in Neuroscience and Behavior, University of New Hampshire, Durham, NH, United States; ^2^Missouri Institute of Mental Health, University of Missouri-St. Louis, Berkeley, MO, United States; ^3^TIRR Memorial Hermann, Department of Neuropsychology, Houston, TX, United States; ^4^Department of Neurology, F. Edward Hebert School of Medicine, Uniformed Services University of the Health Sciences School of Medicine, Bethesda, MD, United States

**Keywords:** mild traumatic brain injury, fatigue, effort, functional connectivity, fMRI

## Abstract

Mental fatigue in healthy individuals is typically observed under conditions of high cognitive demand, particularly when effort is required to perform a task for a long period of time—thus the concepts of fatigue and effort are closely related. In brain injured individuals, mental fatigue can be a persistent and debilitating symptom. Presence of fatigue after brain injury is prognostic for return to work/school and engagement in activities of daily life. As such, it should be a high priority for treatment in this population, but because there is little understanding of its behavioral and neural underpinnings, the target for such treatment is unknown. Here, the neural underpinnings of fatigue and effort are investigated in active duty military service members with mild traumatic brain injury (mTBI) and demographically-matched orthopedic controls. Participants performed a Constant Effort task for which they were to hold a pre-defined effort level constant for long durations during fMRI scanning. The task allowed for investigation of the neural systems underlying fatigue and their relationship with sense of effort. While brain activation associated with effort and fatigue did not differentiate the mTBI and controls, *functional connectivity* amongst active brain regions did. The mTBI group demonstrated immediate hyper-connectivity that increased with effort level but diminished quickly when there was a need to maintain effort. Controls, in contrast, demonstrated a similar pattern of hyper-connectivity, but *only* when maintaining effort over time. Connectivity, particularly between the left anterior insula, rostral anterior cingulate cortex, and right-sided inferior frontal regions, correlated with effort-level and state fatigue in mTBI participants. These connections also correlated with effort level in the Control group, but only the connection between the left insula and superior medial frontal gyrus correlated with fatigue, suggesting a differing pattern of connectivity. These findings align, in part, with the dopamine imbalance, and neural efficiency hypotheses that pose key roles for medial frontal connections with insular or striatal regions in motivating or optimizing performance. Sense of effort and fatigue are closely related. As people fatigue, sense of effort increases systematically. The data propose a complex link between sense of effort, fatigue, and mTBI that is centered in what may be an inefficient neural system due to brain trauma that warrants further investigation.

## Introduction

A signature injury of service members deployed during the conflicts in Iraq and Afghanistan is traumatic brain injury (TBI). Of the approximately 360,000 service members who suffer from TBI, 70% are classified as mild injuries (mTBI; DVBIC Quarterly Reports). At least 19% of the service members with mTBI have persistent symptoms that contribute to difficulty engaging in social and work activities. The consequences of persistent fatigue in mTBI pose a real challenge to rehabilitation (1). High levels of mental *fatigue* commonly persist and relate to failure to return to work and loss of productivity (2, 3). In fact, presence of fatigue is the strongest predictive factor of poor outcomes following TBI (1). Despite the prevalence of fatigue in TBI, our understanding of its behavioral and neural underpinnings is lacking.

Mental fatigue is a complex process that is operationally defined by time on task and increased mental effort. When performance suffers (reaction time, accuracy, etc.) over time, presumably from fatigue, there tends to be fairly diffusely increased brain activity (4). Simultaneously, there may also be decreased motivation under high *effort* (5). According to Kahneman's “resource capacity theory,” the amount of effort needed to perform a task is related to the complexity of the task and an individual's limited general capacity to perform mental work [i.e., resource capacity, (6, 7)]. When a task is difficult, the demand for resources is high, and performance suffers when resources near depletion. When a person recognizes that performance is suffering, tasks are perceived as more difficult, and require greater effort, which Kahneman equates with the experience of mental fatigue.

Brain imaging in mTBI indicates an increase in brain activity with increased time on task regardless of the type or demand requirements of the task (8). In contrast, healthy individuals have decreased activation over time without a serious decrement in performance, and without reporting significant fatigue. This brain response in TBI may suggest a perception of higher levels of effort when the task is long, or that individuals with TBI inefficiently regulate cognitive control and exert more mental effort to maintain a high-level of performance, resulting in fatigue.

While there is a plethora of literature reporting that task demand causes degradation of performance in mTBI, few have investigated whether task demand results in fatigue more so than in healthy controls, or how this fatigue manifests in behavior or in neural function. The few available studies have small sample sizes [e.g., (9)] limiting their generalizability. The brain networks implicated in effort and fatigue include frontostriatal circuitry, or the ventromedial prefrontal cortex more specifically. Damage to these brain regions is thought to diminish resource capacity and impair allocation of resources, resulting in an increased perception of expended effort (10–12). Additionally, fatigue related to lack of motivation to engage and maintain performance on a task, or to predict and manage change in performance based on feedback about performance, is associated with the integrity of the ventromedial prefrontal cortical. That is, individuals with larger lesions of this brain region report more fatigue and apathy (13, 14). The frontostriatal network is involved in coding the incentive value for an expected outcome (15), and is mediated by dopaminergic frontostriatal networks (13, 16–19). Breakdowns in ventromedial prefrontal cortex-related network connectivity may disrupt the ability to appropriately detect, monitor, and self-correct errors or to adequately motivate behavior (20, 21). For example, the anterior cingulate cortex is associated with monitoring and detecting errors, the pre-supplementary motor area with engaging in task, and the connectivity amongst these two regions is related to fatigue (22).

One gap in the existing literature on fatigue is that paradigms infer “probable” fatigue [exception is Wylie et al. (22)], rather than directly measuring it. In the present study, we investigate brain activity and network connectivity in mTBI participants while they perform a task explicitly designed to study the relationship between task-related effort and fatigue. We assess fatigue with a questionnaire about fatigue over the week prior to scanning (trait) as well as with task manipulation during brain imaging [state, Constant Effort Task [CE]]. For Constant Effort, subjects are asked to squeeze a bulb to a prescribed *effort* level and hold it constant for a discrete period of time. The task is considered a general index of central fatigue as it is not specific to motor system engagement (23, 24). Varying effort levels result in predictable changes in the ability to maintain pressure on the bulb such that the time it takes to fatigue is slower at low effort levels than at higher effort levels. Performance on the CE task during functional fMRI allowed for identification of the neural systems underlying effort and fatigue as well as the differences in these systems in mTBI relative to control. We hypothesize that fatigue in mTBI arises when there is an altered perception of the amount of effort needed to perform the task, either because there is a failure to:

- update the amount of effort given to the task based on internal feedback about performance, which is assessed by contrasting performance across effort levels,
- sustain a given effort level, which is assessed via time on task, or
- both.

Because estimating and maintaining effort are likely a result of a complex network of interacting brain regions, we examined not only brain activation during task performance, but also functional connectivity (FC) amongst the regions active during the task. We predict that mTBI participants will demonstrate increased pre-frontal and anterior cingulate cortex activation, as well as increased connectivity of these regions to ventral-striatal regions relative to Control participants.

## Method

### Materials and Methods

#### Participants

Participants were recruited from consecutive patient referrals to the TBI Service at the San Antonio Military Medical Center in San Antonio, Texas. Written informed consent was obtained for each participant following an explanation of the research with an approved and monitored Institutional Review Board (No. 3743378) and Human Research Protection Office at the U.S. Army Medical Department Medical Research and Material Command (No. A-17660).

mTBI was defined by the VA/DoD Clinical Practice Guidelines for the Management of Concussion/mild TBI (25). Injuries occurred during deployment in support of OEF/OIF and were within 3–24 months of study enrollment. All mTBI participants complained of cognitive symptoms [endorsing symptoms in at least 3 of the 4 cognitive clusters [somatic, sensory, affective, cognitive] on the Neurobehavioral Symptom Inventory (26)]. All participants understood and communicated in English. Participants were excluded if they reported any of the following: blindness/low vision, uncontrolled seizure disorder, psychosis, history of moderate, or severe TBI or penetrating brain injury, spinal cord injury limiting use of upper extremities, were participating in any other intensive treatments (>5 appointments/week) or were on scheduled narcotic pain medications.

### Data Acquisition

*Fatigue Severity Scale* [FSS, (27)] is a nine-item self-rated scale examining the impact of fatigue on motivation, activity level, and social participation. This instrument documents the presence and severity of fatigue over the week prior to assessment and therefore is a metric of trait fatigue.

#### Constant Effort (CE) Task

Participants performed a behavioral task designed to be a metric of sense of effort and a continuous, on-line measure of fatigue (12, 28). CE trials result in pressure-by-time curves usually characterized by an exponential decay (index of fatigue) to a non-zero asymptote. Prior to the scanning session, participants were familiarized with the experimental protocol, performing the task at least once. To establish the maximum level (Pmax) of effort in kilo-pasquals (kPa), subjects were asked to squeeze a pneumatic bulb (IOPI, www.IOPImedical.com) with their right hand as hard as they could. The Pmax is displayed digitally by an LED display on the IOPI, and output to a computer via a custom-designed hardware interface (18.4 mV/kPa amplification; A:D 8-bit conversion; 88 Hz sampling rate). During the task, they squeezed the bulb to a prescribed level of effort (either 25, 50, or 75% of their Pmax) by matching their effort to a display to achieve the desired starting level (see Supplementary Figure 1) for 5 s, and to maintain that level of effort constant for 30 s, according to the method published previously (28). The subject is then given 60 s of rest before the next trial is presented. Trials progressed from the easiest (25% effort) to the hardest effort level (75% effort) for all participants, with two trials performed at each level. The curves are fitted to the equation: F(t) = exp(b - a^*^t) + c where *a* represents the rate of pressure decay, *c* is the asymptote or residual pressure, and *b* is natural log of the y-intercept [the value of F(t) at *t* = 0]. The time constant (TC), defined as the inverse of the parameter *a* in the fitted exponential equation, is used to characterize the rate of declining pressure early in each trial as effort is held constant. The TC essentially represents the amount of time it takes for the pressure curve to decrease to one-third of its total excursion, which varies by individual and has been shown previously to be faster in TBI than in healthy controls (29).

#### Image Acquisition

Each participant underwent multimodal MRI utilizing a 3 Tesla Siemens Verio Syngo scanner (Siemens Medical Solutions USA, Malvern, Pennsylvania) at a large military treatment facility. A high-quality T1-weighted volumetric image was acquired for inspection of anatomical integrity and for spatial normalization and anatomical localization of functional findings. A total of 176 sagittal 3D MPRAGE slices were acquired with the following parameters: slice thickness = 1.0/0.5, TE/TR = 2.6/2530, FOV = 256mm, voxel size = 1 × 1 × 1 mm, 512 × 512 matrix, flip angle = 7°, SENSE factor 2. A total of 40 axial blood-oxygen-level-dependent (BOLD) echo-planar slices were acquired during performance of the CE task with the following parameters: slice thickness = 3.0/0.3 interleaved, FOV = 240 mm, voxel size = 3.43 × 3.43 × 3.0 mm, TE/TR = 30/2500, flip angle = 90°, foldover direction = AP, fat shift direction = P, SENSE factor 2.0 for a total of 230 images acquired over a 9.6-min continuous scan.

### Data Analysis

#### CE Task: Analysis of IOPI Data

A filtering algorithm was chosen to model the exponential function of the amount of pressure exerted on the bulb at each effort level (25, 50, and 75%). The best solution, which worked for all subjects, was to use a median sliding window. Supplementary Figure 2 shows an example of the original raw data and the filtered output with the appropriate model. Inspection of the figure shows that filtering eliminates the noise and allows for accurate data analysis to proceed.

#### fMRI Data: Pre-processing

The first four dummy scans were removed and then the EPI images were corrected for head movement by affine registration using a two-pass procedure (SPM12, www.fil.ion.ucl.ac.uk/spm). The mean EPI image for each subject was then spatially normalized to the MNI single subject template using “unified segmentation” approach (30). The ensuing deformation field was then applied to the individual EPI volumes and a 5-mm full-width half mass (FWHM) Gaussian kernel smoothed the output images. Finally, the images were spatially smoothed (8 mm, FWHM).

#### fMRI Data: General Linear Model (GLM)

Once the images were pre-processed, a whole-brain analysis was performed to address the hypotheses regarding effects of effort level (25, 50, 75%), half (i.e., first/second), and group (mTBI or control). A block consisted of 10 TRs (25 s) during which a participant was holding an effort level constant. The first 5 TRs within a block were considered the first half of the trial, and the last 5 the second half. Thus, for each effort level, two blocks of 10 TRs were assessed for the effect of effort (total of 20 TRs per level) and the first 5 TRs of each block was assessed as first half (total of 30 TRs for the entire scan, or 10 TRs at each effort level). The independent variables were convolved with a canonical hemodynamic response function, along with six motion parameters included as regressors of non-interest. Significance was defined at *p* < 0.05, corrected for family-wise error, with an extent threshold of *k* > 10 contiguous voxels.

#### fMRI Data: Functional Connectivity (FC)

Regions of interest (ROIs) used in the FC analysis were those meeting statistical threshold for the main effects of effort, half, or group (Supplementary Tables 1, 2, 3). BOLD time-series were sampled at each peak coordinate for a 6 mm sphere for each subject and differentiated for effort and phase. For each subject, we then computed linear (Pearson) correlation coefficients between the extracted time series of each ROI to examine connectivity. These voxel-wise correlation coefficients were then transformed into Fisher's Z values where each score represents the FC strength for each connection in each subject. Significant differences between groups were identified using independent samples *t*-tests (FDR-corrected *p* < 0.05). Within subject, repeated-measures differences in FC were assessed using the GLM analysis (Bonferroni corrected *p* < 0.05) to identify interactions and main effects for effort, phase, and group.

## Results

Thirteen of the 115 participants were excluded from the data analyses due to excess movement during scanning or poor coverage of the structures needed for the FC region of interest analyses. Table 1 is a summary of the demographics and characteristics of brain injury for the group participants. Most TBI events were blast injuries [63% Blast, 20% Other [blunt trauma, flash burns, and one gunshot wound], 10% Falls, and 7% motor vehicle accident] and few participants reported loss of consciousness (*n* = 10; only one for longer than 1 min) or posttraumatic amnesia (*n* = 1). The injuries had occurred at least 60 days prior to participation in the study (mean number of days post onset of injury = 292; standard deviation = 176 days). More mTBI participants endorsed symptoms of chronic pain, depression, and anxiety than the Control group, but group differences were not significant for *current* diagnosis (Table 2). The mTBI group also reported higher fatigue severity on the FSS (mTBI mean = 39, standard deviation = 12; Control mean = 25, standard deviation = 10; *t*_99_ = 5.93, *p* < 0.0001).

**Table 1:** The mTBI and Control groups did not significantly differ for any variable except education level^a^ [$\chi_{\left(4\right)}^{2}$ = 9.5, *p* = 0.049], with the mTBI group having more post-high school education.

	**mTBI**	**Orthopedic control**
*n*	60	42
Age	36 ± 8	33 ± 10
Gender	7F/53M	2F/40M
**EDUCATION**^**a**^		
General Education Diploma	1	4
High School Diploma	22	23
Associate's Degree	14	7
College Degree	14	3
Post Graduate	9	5
RACE^*^		
Asian	1	0
American Indian or Alaska Native	1	2
Black or African-American	13	6
More than One Race	3	1
Native Hawaiian or Other Pacific Islander	0	1
White	42	30
**ETHNICITY**		
Hispanic or Latino	18	9
Not Hispanic or Latino	42	33
Marital Status		
Single, Never Married	14	7
Married	42	26
Separated	0	3
Divorced	4	6
**MILITARY BRANCH**		
Air Force	9	1
Army	49	38
Navy	1	2
Marine Corps	1	1
Years Served^c^	14 ± 8	11 ± 8
# of Deployments	2 ± 1	2 ± 1

* *Missing Race data for one participant*.

**Table 2:** Numbers of participants having current comorbid conditions did not differ between groups; however, rates were slightly higher in the mTBI group.

	**mTBI**		**Orthopedic control**	
Chronic pain	27		15	
Amputation	2		5	
	Current	Prior	Current	Prior
Depression	22	9	18	4
Anxiety^a^	28	14	20	2
Prior ADHD	4	7	4	5
PTSD	25	8	19	3
Learning disability^b^	0	0	2	4
Substance abuse	0	0	0	0
Alcohol abuse	0	3	1	2

a *mTBI > Control for prior anxiety [$\chi_{\left(1\right)}^{2}$ = 6, p = 0.011]*.

b *Control > mTBI for prior learning disability [$\chi_{\left(1\right)}^{2}$ = 6, p = 0.015]*.

### Constant Effort Task Results

Task performance data acquired in the MRI scanner suffered from magnet-related noise affecting the ability to obtain quality CE data for several of the subjects. Analyses for participants with adequate data (mTBI 25% Effort *n* = 28, 50% Effort *n* = 38, 75% Effort *n* = 23; Control 25% Effort *n* = 26, 50% Effort *n* = 36, 75% Effort *n* = 34) yielded no group differences in Time Constant at any effort level (all *p*s >0.5; Supplementary Figure 3).

### fMRI Task-Related Activation Results

#### Effect of Time on Task

**Figure 1:**
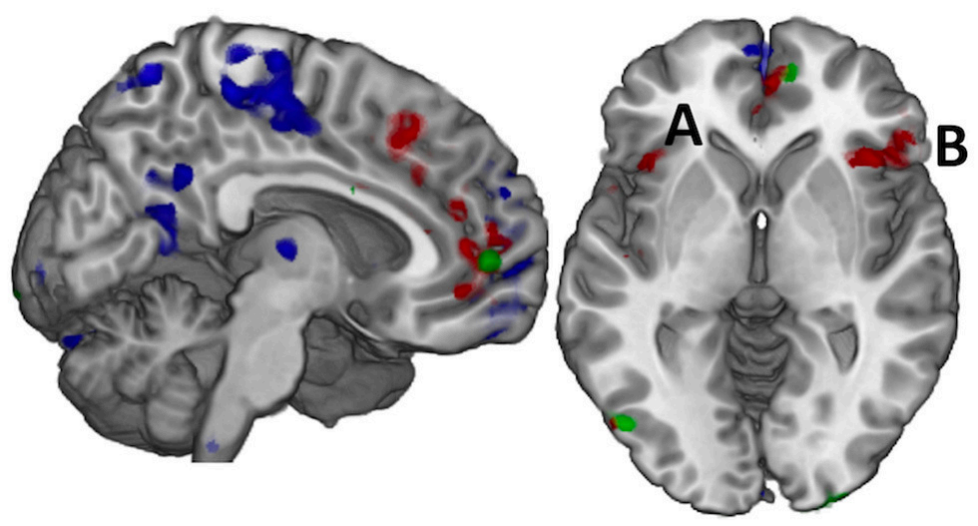
Effort and Fatigue in the Constant Effort task demonstrated differing regional effects with effort associated with caudal, medial prefrontal cortex (red) while fatigue was associated with rostral prefrontal cortex as well as postcentral and posterior cingulate cortex (blue). Controls demonstrated significantly higher activity than mTBI in a small area of the right medial prefrontal cortex (green) while mTBI had more activity in the posterior occipital cortex, but there were no other significant group effects. When these regions were used in computing functional connectivity, it was only the connectivity amongst the regions of the effort effect (red) that demonstrated group differences in connection strength. For example, the connection between the left insula **(A)** and the right inferior frontal gyrus (**B**, pars orbitalis) was significantly stronger in the TBI group for time on task at 75% effort.

As participants sustained pressure on the IOPI bulb at each effort level, a main effect of fatigue was observed as the change in brain activation from the first to the second half of each trial. This effect was robust in frontal, parietal, and cerebellar regions (blue in Figure 1, Supplementary Table 1). *Post hoc* analysis of directional effects identified that bilateral activity of the lateral superior and inferior parietal cortex and cerebellum was greater in the first half of each trial relative to the second, but there was more distributed and robust activity of the medial pre-frontal and medial parietal cortex in the second half relative to the first.

#### Effect of Effort Level

**Figure 2:**
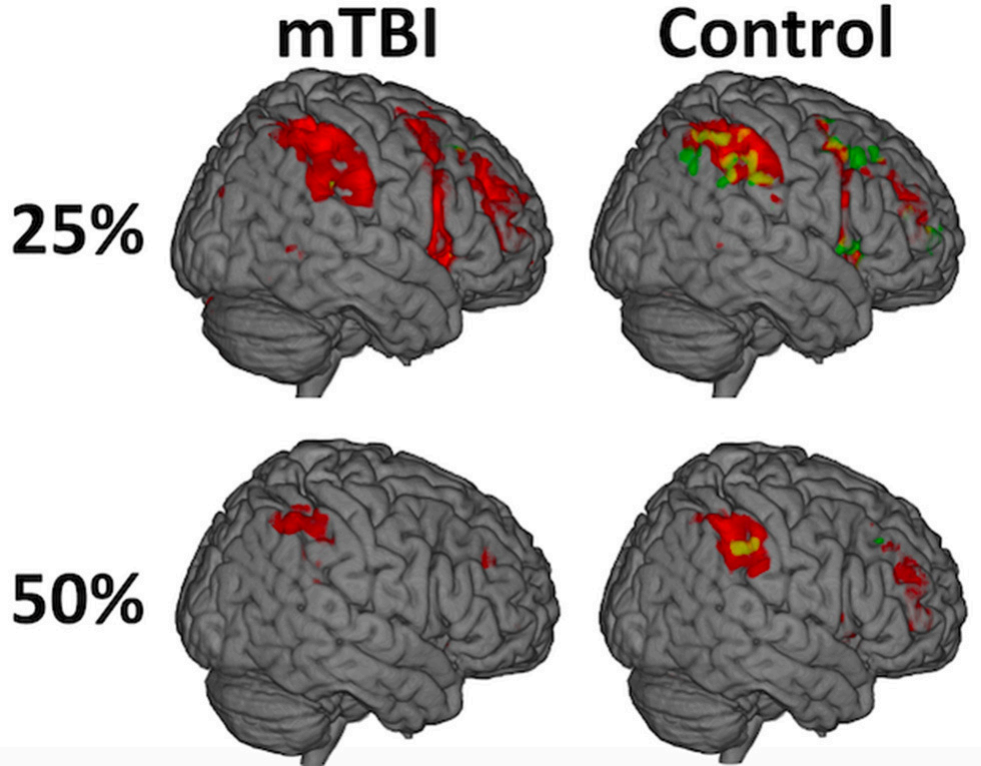
While there was not a significant Time on Task-by-group interaction (A = mTBI; B = Control), the distribution of activity during the first half (red) relative to the second half of each trial (green) differed between the groups and was most evident at the 25% effort level such that the mTBI group had little to no suprathreshold activity in the second half of each trial while the Control group did. The distribution of the effect, and the amount of brain activation, decreases over the duration of the experiment (e.g., from 25 to 50%) in both groups and is only seen in the left postcentral gyrus at 75%, likely secondary to habituation to the ask over time.

A main effect of effort level was observed primarily in the right hemisphere in anterior insula, inferior frontal, and cingulate cortex as well as in the middle temporal and supramarginal gyri (red in Figures 1 and 2, Supplementary Table 2). Activity was greater in the 25% effort condition relative to the other two effort conditions.

#### Effect of Group

A main effect of group was observed in a small area of the superior medial gyrus (Control > mTBI) and in the visual cortex (mTBI > Control; Figure 1, Supplementary Table 3).

#### Interaction Effects

**Figure 3:**
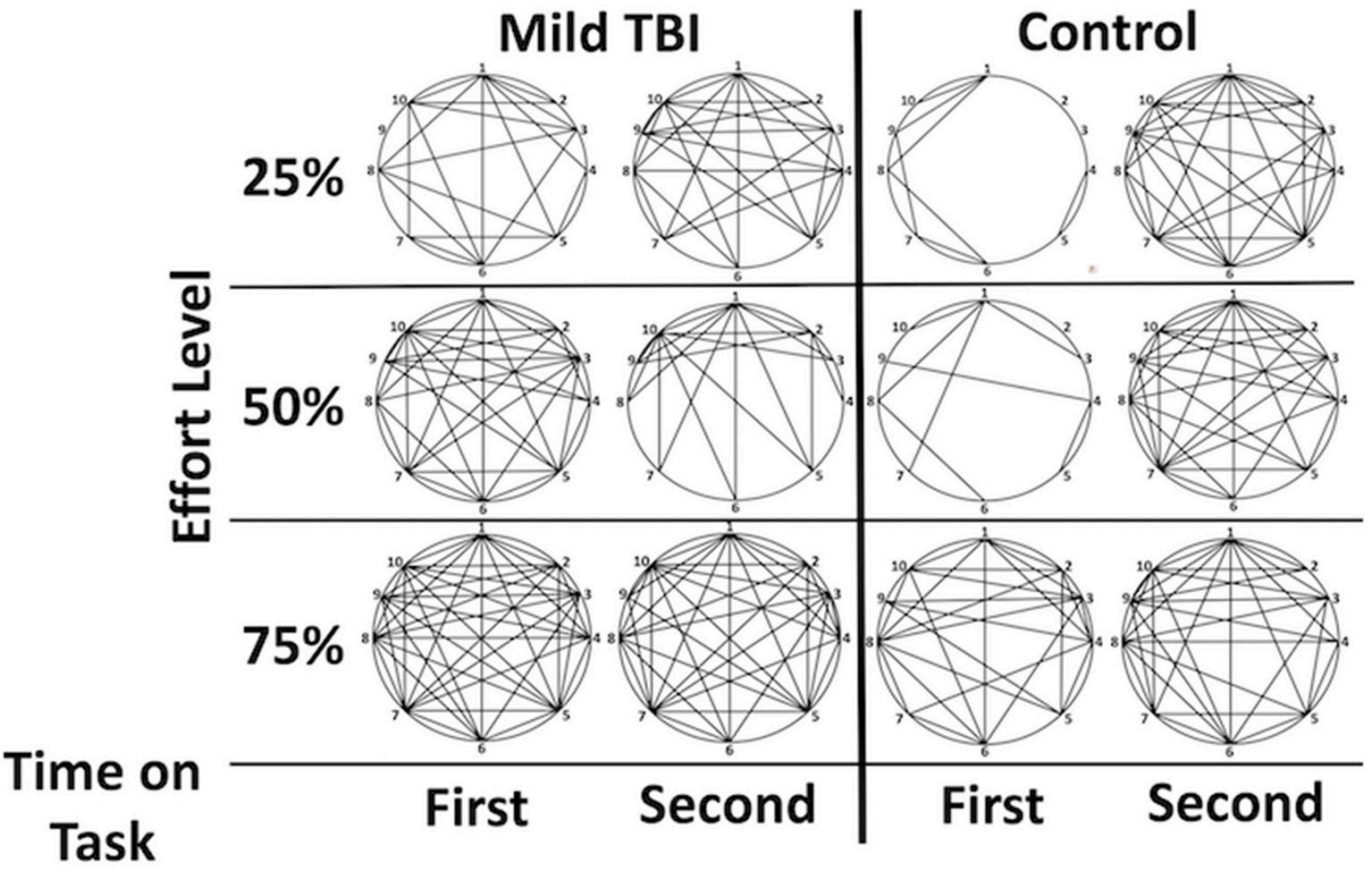
Graphical demonstration of functional connectivity between the Effort regions by group and phase highlights the (1) increased connectivity in the mTBI group and the relative difference between phases one and two in the control group that is not as evident in the mTBI. Lines indicate a functional connection between two regions at z > 3. Correlations between functional connectivity strength and time post injury were minimal but existed between the 2 right insular regions for the second half of the 25% effort level trials (*r* = −0.372), between the left and right insula for the first half of the trials at 75% (−0.302 for right insula; −0.374 for right insula F03). Legend: 1 = dorsal ACC, 2 = rostral ACC, 3 = right inferior frontal operculum, 4 = right insula (orbitofrontal), 5 = right insula, 6 = left postcentral gyrus, 7 = left insula, 8 = left precentral gyrus, 9 = right inferior frontal gyrus (p. orbitalis), 10 = superior medial frontal gyrus.

While there was not a significant *Time on Task-by-group interaction*, the distribution of activity during the first half of a trial relative to the second half differed between the groups, most strongly in the 25% effort level such that the mTBI group had little to no suprathreshold activity in the second half of the trials while the Control group did (Figure 3, Supplementary Table 4). The spatial distribution of this temporal effect decreased over the duration of the experiment (e.g., from 25 to 50%) in both groups and is only seen in the left postcentral gyrus at 75% in Controls.

Interaction effects for Time on Task-by-Effort Level or for Effort Level-by-Group did not meet statistical significance.

### Functional Connectivity Results

#### Group Differences in FC

**Figure 4:**
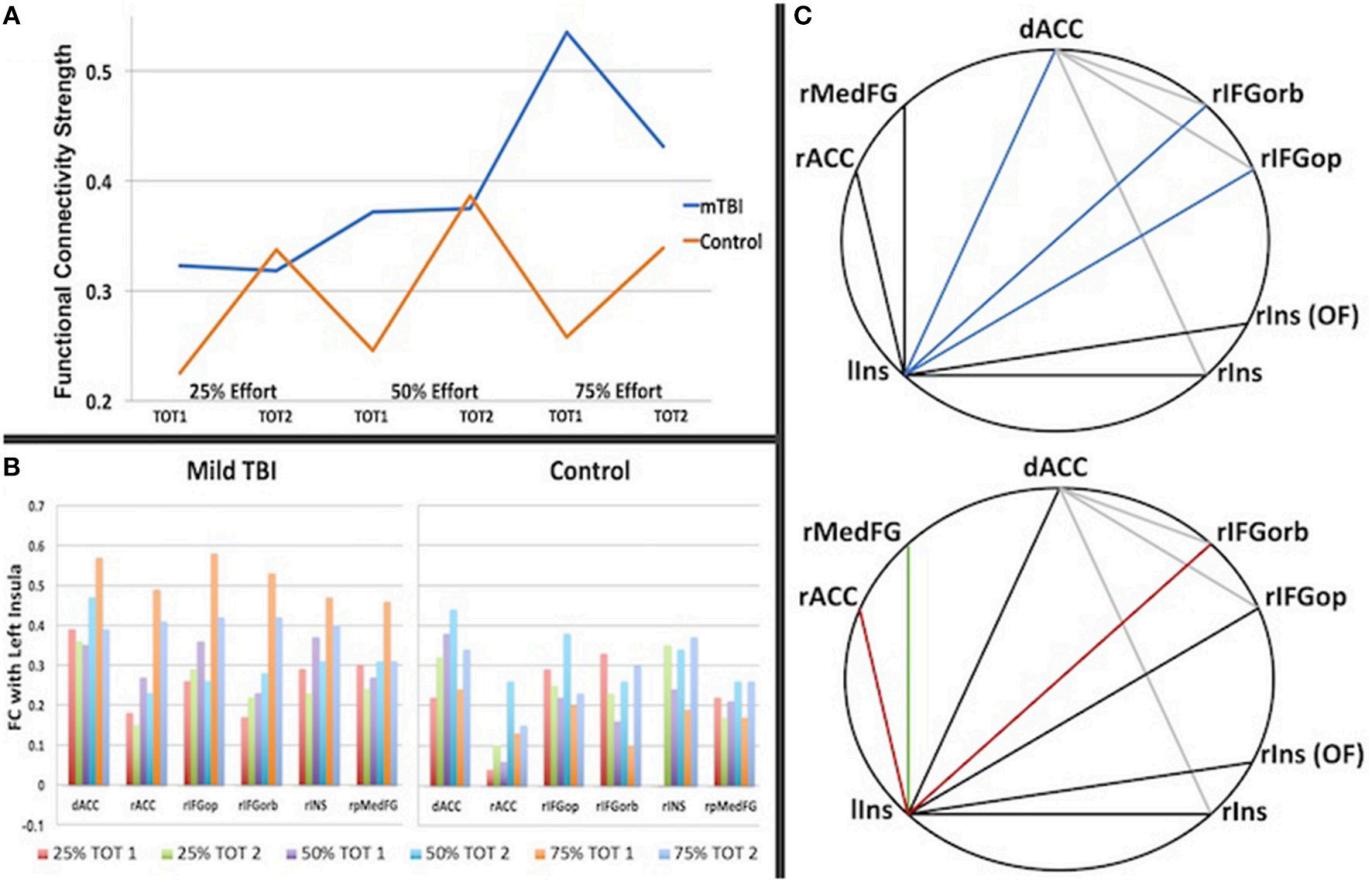
The graph in **(A)** is a summary of the strength of connectivity across all region-to-region connections for Time on Task (TOT) in which there was a significant group difference (listed in Table 3). It illuminates the different patterns of connectivity change with effort and TOT, with the Control group showing an increase in connectivity strength in the second half of each trail at each effort level whereas the mTBI group does not show a TOT difference in functional connectivity strength at 25 or 50% but a steep increase in strength in the first half of each trail at 75% effort relative to the second half. In **(B)**, the differences are also evident by region in functional connectivity for the first half of the 75% -phase 1 condition (orange), particularly in the connections with other regions with the left insula. **(C)** Demonstrates the inter-regional connections that differed between the groups, with the darker lines representing connections with the left insula and colored lines indicated correlation of that connection with the trait (FSS, top) or state (CE Time Constant, bottom) measures of fatigue. (**C**, top): Blue = connection correlates with the FSS for the first half of the 75% effort trials (left insula-dorsal ACC, rho = 0.28, p = 0.004; left inusula-right frontal orbitalis, rho = 0.25, *p* = 0.01, left insula-right frontal operculum, rho = 0.22, *p* = 0.027). (**C**, bottom): Red = connection correlates with the CE Time Constant for the first half of the 75% effort level (left insula-rACC rho = −0.35, *p* = 0.023 for all subjects; rho = −0.41, *p* = 0.04 for mTBI group; left insula-right frontal orbitalis rho = −0.57, *p* = 0.003 for mTBI group); Green = connection correlates with the CE Time Constant for the first half of the 75% effort level trials in the Control group (rho = −0.58, *p* = 0.012).

Significant group differences in FC were only observed amongst the brain regions in which there was an effect of effort level (henceforth ROIs, Supplementary Table 2). FC tended to be stronger amongst the ROIs in the mTBI relative to the Control group across all effort levels (Figure 4), particularly in the 50 and 75% effort levels and specifically in the first half of each trail, indicating that connectivity amongst regions associated with the manipulation of effort level distinguished the groups. For the first half of each trial at the 50% effort level, the mTBI group had stronger FC between the right posterior medial gyrus and: (1) the dorsal anterior cingulate cortex [*t*_(100)_ = 3.12, *p*_FDR_ = 0.045] and (2) the left postcentral gyrus [*t*_(100)_ = 3.123, *p*_FDR_ = 0.045] as well as between those two in the right insula ROIs [*t*_(100)_ = 3.19, *p*_FDR_ = 0.045]. Larger group differences were observed in the first half of each trial in the 75% effort level (Figure 3), again with stronger FC in the mTBI group relative to the Control group, and centering on left insula connectivity with several of the other ROIs (Figure 4).

**Table 3:** Functional connectivity values between the ROIs at each effort level by group.

	**25% Effort**				**50% Effort**				**75% Effort**			
	**Phase 1**		**Phase 2**		**Phase 1**		**Phase 2**		**Phase 1**		**Phase 2**	
	**mTBI**	**C**	**mTBI**	**C**	**mTBI**	**C**	**mTBI**	**C**	**mTBI**	**C**	**mTBI**	**C**
Dorsal ACC _ Left Insula	0.39	0.22	0.36	0.32	0.35	0.38	0.47	0.44	0.57^*^	0.24	0.39	0.34
Dorsal ACC–Right Frontal Operculum	0.4	0.2	0.26	0.45	0.41	0.37	0.41	0.52	0.59^*^	0.32	0.39	0.34
Dorsal ACC–Right Insula	0.4	0.15	0.33	0.31	0.33	0.21	0.37	0.4	0.47^*^	0.16	0.39	0.29
Dorsal ACC–Left Frontal Orbitalis	0.23	0.3	0.22	0.33	0.22	0.24	0.35	0.3	0.44^*^	0.16	0.32	0.32
Dorsal ACC–Right Medial Gyrus	0.6	0.59	0.81	0.71	0.81^*^	0.54	0.72	0.79	0.87	0.77	0.79	0.67
Left Insula – Rostral ACC	0.18	0.04	0.15	0.1	0.27	0.06	0.23	0.26	0.49^*^	0.13	0.41	0.15
Left Insula _ Right Frontal Operculum	0.26	0.29	0.29	0.25	0.36	0.22	0.26	0.38	0.58^*^	0.2	0.42	0.23
Left Insula _ Right Insula	0.29	−0.002	0.23	0.35	0.37	0.24	0.31	0.34	0.47^*^	0.19	0.4	0.37
Left Insula _ Right Insula (OF)	0.2	0.13	0.16	0.44	0.23	0.32	0.27	0.29	0.44^*^	0.13	0.37	0.21
Left Insula _ Left Frontal Orbitalis	0.17	0.33	0.22	0.23	0.23	0.16	0.28	0.26	0.53^*^	0.1	0.42	0.3
Left Insula _ Right Medial Gyrus	0.3	0.22	0.24	0.17	0.27	0.21	0.31	0.26	0.46^*^	0.17	0.31	0.26
Right Insula _ Right Insula (OF)	0.46	0.36	0.5	0.44	0.61^*^	0.2	0.53	0.56	0.76	0.52	0.65	0.56
Left Postcentral _ Right Medial Gyrus	0.32	0.1	0.37	0.29	0.37^*^	0.04	0.36	0.22	0.29	0.26	0.35	0.37

* *Group differences, p < 0.05*.

#### Functional Connectivity and Measures of Trait and State Fatigue

There were some minimal correlations between FC and the FSS or CE Time Constant data for each group as follows.

##### Fatigue severity scale and functional connectivity

As demonstrated in Figure 4C (top) FSS self-report scores for all participants correlated with FC between the left insula and the dorsal anterior cingulate cortex (rho = 0.28, *p* = 0.004), the left insula and the right inferior frontal gyrus (pars opercularis, rho = 0.255, *p* = 0.01; pars orbitalis, rho = 0.22, *p* = 0.027), as well as the between the dorsal anterior cingulate cortex and the right inferior frontal gyrus (pars orbitalis, rho = 0.22, *p* = 0.03) during the first half of the 75% effort level trials.

##### Constant effort time constant and functional connectivity

As demonstrated in Figure 4C (bottom), FC between regions correlated with the CE Time Constant for the first half of the 75% effort level in both groups, centering again on connectivity with the left insula, but with group differences in the regional connections demonstrating this relationship. That is, the mTBI group demonstrated a moderate negative correlation between 75% effort TC and left insula and right inferior frontal orbitalis FC (rho = −0.41, *p* = 0.04) whereas the Control group demonstrated a moderate negative correlation between 75% effort TC and left insula-right superior medial frontal gyrus (rho = −0.58, *p* = 0.012). Both groups combined demonstrated a minimal correlation between 75% effort TC and left insula-rostral anterior cingulate FC (rho = −0.35, *p* = 0.023).

## Discussion

In this study, there were three main findings about effort and fatigue in mTBI. First, the mTBI and Control groups did not show differences in effort-related *activation* aside from slight differences in activation for Time on Task (Figure 1). That is, the mTBI group sustained a consistent level of activity for less time per CE trial than the Control group. Second, the mTBI group demonstrated *hyper-connectivity* at all effort levels amongst the effort-related ROIs (Figure 3). And third, FC of the left insula with other effort-related ROIs was hyper-connected in the mTBI group during the first half of each trial and decreased in the second half, a pattern *opposite* of that observed in the Control group.

If the Control group can represent a model for how FC should map to the CE task, then it appears that effort level for this task should not result in elevated FC, but time on task should. The opposite, however, was observed in the mTBI group with elevated FC at each effort level and not with time on task except during the most difficult effort level (75%). Because our measures of fatigue correlated with FC during the 75% effort level (Figure 4C and Supplementary Table 6)–the condition that differed most between groups, we postulate that FC amongst all the ROIs is sensitive to trait and state fatigue. It follows then, that the FC data support our hypothesis that mTBI participants misevaluate effort levels and fail to update their effort levels based on internal feedback about their performance, which results in fatigue.

Previous studies of effort in TBI have been in more severely impaired participants than those in the present study, but have also reported hyperactivity that has been interpreted as evidence of increased effort (31). The lack of significant differences between the groups in task-related activation in the present study may either be related to the mild nature of brain injury or to the lack of cognitive demand or mental load in the Constant Effort Task. Scheibel et al. also report increased activity with Time on Task in their more severely impaired TBI group whereas we report a reduction in activity over time in the mTBI group (32). Our interpretation of this lack of group difference in *activation patterns* is that the participants in each study differed in severity of impairment, the tasks differed between studies, and that our mTBI group was not as mentally taxed by our task and were able to perform at near-normal levels.

The apparent difference between the groups in the present study was in the *strength of functional connectivity* amongst the regions in which activity was modulated by effort level. The mTBI group demonstrated heightened connectivity across all effort levels whereas the Control group did not evidence elevated connectivity until performing at the 75% effort level (Figures 3, 4A).

The other major difference between groups was an overall increase in functional connectivity in the mTBI participants. Hyper-connectivity in TBI has been reported in resting-state (33, 34) and task-related brain function (35). Animal models of closed head, controlled cortical impacts have posed that functional hyper-connectivity is common after injury and may represent cortical reorganization at local and diffuse levels (36). However, this reorganization does not mirror the time line of recovery from structural damage (i.e., axonal reorganization, sprouting) and rather may relate to a glutamate/GABA imbalance that occurs with brain tissue recovery (37). Hyper-connectivity persists at 28-days post injury in the rat model, but there are few data that can speak to the longitudinal change in hyper-connectivity, if any, in humans with TBI. Time post injury in the cohort presented here showed only minimal negative correlation with functional connectivity (see Supplementary Table 5), but all participants were more than 60-days post injury. As such, this phenomenon, and how it may change over time with recovery, continues to be poorly understood.

Importantly, this is among the first studies to report that hyper-connectivity in the mTBI group was altered with increasing effort level, suggesting that though hyper-connectivity may be a physiological response to injury, it is moderated by task demands. Connectivity also varied with effort level in the Control group, but the pattern of connectivity variance with effort level was very different between the groups (Figure 4A). Increasing connectivity strength amongst the ROIs was followed by a steep decrease in connectivity with time on task, particularly for the 75% effort level, in the mTBI group. The Control group, in contrast, had only a minimal increase in functional connectivity with effort level but a striking increase in connectivity with time on task. We interpret the Control data as suggesting that functional connectivity amongst these ROIs is essential to *sustaining* performance at higher effort levels, but not for estimating effort or initiating performance. The mTBI participants engage that higher degree of connectivity strength simply to initiate effort, even at low effort levels, but appear unable sustain that level of connectivity when they begin to fatigue in the second half of a trial.

While many other investigators have explored brain activity in TBI during tasks that are complex and with high degrees of mental effort, none have investigated whether participants can put forth a set amount of effort and sustain it. Here, we have been able to model the decline in effort and have a quantifiable measure of fatigue (CE Time Constant). We note that one recent investigation contrasted two levels of the n-back task (0-back: not effortful; 2-back effortful) as well as a visual analog scale before and after each to measure state fatigue in TBI and control participants (38). Fatigue in that experimental context was associated with increasing caudate nucleus activity in the TBI participants in the 2-back condition (effortful); the Control participants had decreased caudate activity. Activity of the caudate was linked by these authors to the dopamine imbalance hypothesis such that TBI participants demonstrate more activation of regions of the dopaminergic brain networks when motivated to engage in a task (16, 39). The imbalance in the case of TBI is that fatigue results when the motivation to engage in the task is outweighed by the effort required, indicating an interaction between fatigue and effort that moderates the dopaminergic system. This finding in the caudate nucleus was not replicated in the present study, though we did find subthreshold caudate nucleus activity (at *x* = −14, *y* = 22, *z* = 10, *F* = 11, *p*_*uncorrected*_ < 0.05) in an effort level-by-group interaction such that there was higher activity in the mTBI group at the 50% effort level while the Control group had higher activity at the 75% effort level. This may suggest a role of the caudate nucleus region in effort level, but not in time on task.

Wylie et al. also reported that caudate activity was positively correlated with their state measure of fatigue (visual analog scale before and after the n-back task) validating its role in cognitive fatigue (38). Our state (CE Time Constant) and trait (FSS) measures of fatigue correlated with connectivity strength between the left anterior insula and medial frontal brain regions during the first half of the 75% effort trial, with the difference being that the dorsal anterior cingulate connections correlated only with the trait measure. Thus, we speculate that these data suggest a central role of these structures and their connectivity in fatigue, but are cautious because our CE Time Constant data was missing for several participants.

Although our data did not point to activity of caudate nucleus or other dopaminergic network activity with effort or fatigue, there was an obvious link between other regions of the effort-related network and the *anterior insula*. The anterior insula is increasingly active during tasks requiring cognitive effort (40, 41) and proposed as an essential hub for efficiency of cognitive ability (42) in concert with the anterior cingulate cortex. That is, the anterior insula-to-anterior cingulate co-activity decreases in individuals who perform well and sustain performance under higher cognitive load – i.e., become more efficient (43). It is unclear how these findings reconcile with ours of hyper-connectivity of the insula with, among others, the rostral anterior cingulate and therefore further study is warranted. However, that this connection also correlated with state and trait measures of fatigue suggests that it may be central to the link between effort and fatigue.

We recognize limitations to the present study. For example, the presence of comorbidities in the mTBI group may have influence on the findings in the present study. Future investigation into the effects of comorbid chronic pain, posttraumatic stress disorder, and depression, which are more common in mTBI than that observed in orthopedic control participants (44, 45), on fatigue and effort is warranted. Another common comorbid condition in mTBI is sleep disturbance, which was not assessed in the present study. For our purposes, the fatigue that may arise from sleep difficulties, is considered “trait” fatigue. In fact, the FSS has been shown previously to correlate with sleep quality [e.g., (46)]. The fatigue assessed with the Time Constant, i.e., that elicited through the task manipulations, is considered “state” fatigue. Time Constant did not correlate with the FSS, suggesting that the fatigue associated with CE task performance is a construct unrelated to sleep. Again, however, further study investigating the effects of sleep quality and quantity on effort and fatigue in mTBI is warranted to clarify. And finally, we note that brain injury does frequently increase variability in performance along a number of parameters (47). Thus, it is possible that even with the instruction to hold effort constant, there was variability associated with the output of the CE task. The purpose of this task was to examine a single parameter of central fatigue/sense of effort during brain scanning and this is what drove our hypotheses. It may be important in future work to determine an index of variability and study this in relation to brain injury, effort and fatigue.

## Conclusions

In sum, our findings suggest that brain activity during Constant Effort does not differentiate mTBI and Control participants, but that FC amongst regions associated with effort level does. FC in mTBI participants elevates incrementally with effort level such that all regions are co-active to *initiate* performance, but this co-activation diminishes quickly. FC in Control participants also elevates with effort level but appears to engage in such a way only when there is a need to *sustain* performance—or when fatigue sets in. These data, in part, support our hypothesis that connectivity of medial prefrontal and anterior cingulate cortex to ventral-striatal regions would be higher in mTBI relative to Controls, with the focus of these connections being in the left insula. While a limitation of the present study is a lack of adequate Constant Effort IOPI data to demonstrate a relationship between these findings and that of the time constant as an index of fatigue, the connection between left insula and the rostral anterior cingulate cortex (i.e., a medial prefrontal cortex) was correlated with our behavioral index of fatigue at the 75% effort level, suggesting that the increase in sense of effort in mTBI participants co-occurs with fatigue. Future study is warranted to more firmly establish this relationship.

## Author Contributions

AR, JL, and DR developed the methodology and designed the experiment and analysis. DT recruited and collected the fatigue measures and imaging data at SAMMC. AR and AN processed and analyzed the data. All authors contributed to writing and editing the manuscript.

### Conflict of Interest Statement

The authors declare that the research was conducted in the absence of any commercial or financial relationships that could be construed as a potential conflict of interest.
