# Supplementary material for: Effort and Fatigue-Related Functional Connectivity in Mild Traumatic Brain Injury
Source: Front Neurol. 2019 Jan 18;9:1165. doi: 10.3389/fneur.2018.01165 (PMC6345685; doi:10.3389/fneur.2018.01165)
Supplement: Supplementary file 1 [file Data_Sheet_1.pdf]

Region	Extent (mm3)	Brodmann's Area	x	y	z	F-value	Effect
Frontal							
Precentral Gyrus	8480		42	-24	66	92.15	2nd > 1st
		4a	*12*	*-32*	*72*	*63.23*	
			26	*-26*	*72*	*61.79*	
Postcentral Gyrus			52	-16	56	55.97	
		4a	*16*	*-28*	*72*	*55.66*	
			52	*-10*	*46*	*50.15*	
			58	*0*	*48*	*46.75*	
		4p	*36*	*-24*	*52*	*45.93*	
			32	*-28*	*58*	*41.68*	
Paracentral Lobule			12	-20	78	38.81	
		4p	*36*	*-20*	*44*	*33.91*	
Rectal Gyrus	1520	Fp2	0	56	-16	43.16	2nd > 1st
		Fp2	*-2*	*48*	*-20*	*40.7*	
Mid Orbital Gyrus		*Fp2*	*-2*	*60*	*-10*	*39.56*	
Paracentral Lobule	1512	4a	-2	-24	56	54.68	2nd > 1st
		4a	-2	-34	56	40.5	
			0	-20	62	36.41	
		4a	4	-34	58	34.18	
Posterior-Medial Frontal			4	-20	68	33.08	
Superior Frontal Gyrus	928	Fp1	-16	64	14	42.15	2nd > 1st
Superior Medial Gyrus		Fp2	-2	60	16	38.32	
Superior Medial Gyrus	456	Fp2	-6	64	4	40.54	2nd > 1st
Superior Frontal Gyrus		*Fp1*	*-20*	*64*	*4*	*33.67*	
Rolandic Operculum	296	OP2	36	-20	18	33.83	2nd > 1st
		OP3 (VS)	*38*	*-16*	*18*	*33.4*	
Heschl's Gyrus		*TE 1.1*	*40*	*-28*	*14*	*31.1*	
Middle Frontal Gyrus	256		-26	32	52	34.12	2nd > 1st

Superior Frontal Gyrus			*-20*	*36*	*42*	*32.48*	
			-20	*42*	*48*	*31.3*	
Superior Medial Gyrus	160		0	56	24	32.32	2nd > 1st
Parietal							
Superior Parietal Lobule	2256	7PC	46	-44	56	56.46	1st > 2nd
		7A	*42*	*-54*	*58*	*54.88*	
Inferior Parietal Lobule		*hIP3*	*38*	*-42*	*48*	*34.65*	
Inferior Parietal Lobule	768	1	-50	-38	56	49.83	1st > 2nd
Postcentral Gyrus		*1*	*-38*	*-42*	*64*	*36.08*	
		PFm	*-44*	*-46*	*56*	*33.41*	
Precuneus	720	5L	6	-62	66	52.12	1st > 2nd
		7P	*8*	*-72*	*60*	*33.06*	
Superior Parietal Lobule	88	7A	24	-70	58	29.64	1st > 2nd
Inferior Parietal Lobule	2200	PGp	-46	-76	38	61.33	2nd > 1st
		PGp	-52	-74	30	58.7	
		PGp	-36	-82	42	43.15	
		PGp	-56	-70	20	36.2	
Angular Gyrus		PGp	-50	-66	24	34.96	
		PGp	-48	-82	20	26.81	
Precuneus (PCC)	1024		-8	-58	10	55.34	2nd > 1st
			-2	-56	24	42.56	
			-4	-52	16	32.32	
Paracentral Lobule	688	4a	-8	-32	70	69.55	2nd > 1st
Postcentral Gyrus	336		-54	-10	36	45.86	2nd > 1st
Postcentral Gyrus	128		66	-4	24	33.26	2nd > 1st
			64	-2	20	30.07	
Paracentral Lobule	80		10	-42	68	33.13	2nd > 1st

Cerebellum						
VIIa CrusI	3376	-26	-76	-24	61.79	1st > 2nd
VI		*-38*	*-58*	*-26*	*51.73*	
VIIa CrusI		*-38*	*-68*	*-24*	*48.16*	
VIIa CrusI		*-34*	*-68*	*-24*	*47.2*	
VIIa CrusI		*-4*	*-86*	*-24*	*38.91*	
VIIa CrusI		*-14*	*-86*	*-24*	*37.17*	
VIIa CrusI		*-36*	*-82*	*-26*	*30.77*	
VI		*-12*	*-78*	*-20*	*30.31*	
VI	376	22	-48	-22	36.35	1st > 2nd
		30	*-56*	*-22*	*35.82*	
VI	136	22	-68	-20	36.8	1st > 2nd
VIIa CrusI	80	-50	-66	-28	42.95	1st > 2nd

Supplementary Table 1. The effect of time on task (TOT) demonstrates engagement of relatively discrete regions of the bilateral superior parietal cortex and the cerebellum in the first half, but more robust and distributed activation of medial prefrontal and parietal cortex in the second half of each trial.

Lobe	Region	Extent (mm3)	BA	x	y	z	F-value	Explanation of the effect
**Frontal**								
	Posterior-Medial Frontal	312		2	24	48	22.29	
	Inferior Frontal Operculum	296	44	54	12	22	19.75	
			44	54	10	14	19.59	
	Precentral Gyrus	256		-50	2	36	22	
	Inferior Frontal Orbitalis	152		48	32	-6	19.25	
	Pars Triangularis		45	52	30	-2	18.59	
**Limbic**								
	Anterior Insula/Claustrum	376		30	22	-6	22.46	
				46	22	-4	17.94	
				38	22	0	16.47	
	Anterior Cingulate Cortex	328		2	44	6	21.21	25% > others
	Superior Medial Gyrus			2	48	2	19.37	
	Anterior Cingulate Cortex	88		4	20	40	17.39	
	Anterior Insula	136		-36	16	4	20.21	
				-30	18	6	16.6	
	Insula - orbitofrontal	128	Fo3[1]	30	22	-18	20.88	
**Parietal**								
	Postcentral Gyrus	160	2	-46	-34	48	20.99	

Supplementary Table 2. The main effect of effort level indicates primarily right-sided increased frontal and limbic activity during the Constant Effort Task that is somewhat irrespective of effort level. $p < .05$ corrected for family-wise error. BA: Brodmann's area

1. Amunts, K. & Zilles, K. Architectonic Mapping of the Human Brain beyond Brodmann. *Neuron* **88,** 1086–1107 (2015).

Region	Extent (mm3)	Brodmann's Area	x	y	z	F-value	Effect
Frontal							
Superior Medial Gyrus	144		6	50	2	32.23	Control > mTBI
		Fp2	*10*	*52*	*-2*	*31.22*	
Occipital							
Superior Occipital Gyrus	440	hOc3d (V3d)	-16	-92	24	41.22	mTBI > Control
		hOc4d (V3A)	*-18*	*-92*	*20*	*39.17*	
Inferior Occipital Gyrus	384	hOc1 (V1)	26	-102	-4	36.97	mTBI > Control
		hOc3v (V3v)	*32*	*-98*	*-2*	*35.63*	
		hOc2 (V2)	*30*	*-100*	*-4*	*33.8*	
Calcarine Gyrus		*hOc1 (V1)*	*18*	*-102*	*-6*	*29.43*	
Middle Occipital Gyrus	272	hOc4la	-48	-74	-2	38.63	mTBI > Control

Supplemental Table 3. The groups differed only in a small area of the superior medial gyrus and the occipital lobe. $P_{FWE} < .05$, $k > 10$.

Region	Extent (mm3)	x	y	z	F-value
Parietal					
Inferior Parietal Lobule	336	-36	-44	50	9.63
		-36	*-38*	*44*	*7.88*
Temporal					
Middle Temporal Gyrus	864	52	-66	2	14.37
		52	*-66*	*-6*	*10.63*
		44	*-60*	*0*	*8.07*
Occipital					
Middle Occipital Gyrus	5072	-50	-75	0	14.84
		-30	*-92*	*10*	*14.45*
		-42	*-72*	*0*	*14.35*
		-38	*-80*	*2*	*12.91*
		-28	*-90*	*24*	*12.82*
		-24	*-94*	*16*	*11.33*
		-24	*-90*	*0*	*10.2*
		-38	*-88*	*0*	*10.03*
		-30	*-80*	*14*	*9.86*
Middle Occipital Gyrus	3376	32	-76	32	15.79
		26	*-84*	*10*	*12.31*
		30	*-88*	*12*	*11.94*
		30	*-88*	*22*	*10.92*
		34	*-88*	*18*	*10.8*
		36	*-86*	*20*	*10.62*
		30	*-70*	*26*	*9.7*
		36	*-82*	*10*	*9.65*
		28	*-74*	*44*	*8.42*
		40	*-86*	*4*	*7.95*

Subcortical					
Putamen	360	-18	12	-2	11.21
Effort Level x Group					
Frontal					
Inferior Frontal Gyrus (p. Triangularis)	264	44	34	-2	9.77
		36	32	0	8.81
Inferior Frontal Gyrus (p. Orbitalis)	184	40	34	-14	12.1
Limbic					
Anterior Cingulate Cortex	216	18	44	12	10.2
Subcortical					
**Caudate Nucleus**	**392**	**-14**	**22**	**10**	**10.85**

Supplementary Table 4. Interaction Effects were subthreshold ($p_{uncorrected} < .001$, $k > 10$).

		Trait (FSS)		State (CE Time Constant		25%		50%		75%	
**Subjects**	**Regional Connection**	**rho**	**p**	**rho**	**p**	**1**	**2**	**1**	**2**	**1**	**2**
All Subjects	Right Medial Frontal Gyrus – **Right Insula (OF)**	.20	.044				X				
	Right Medial Frontal Gyrus – **Left Insula**	-.21	.034								X
	Right Medial Frontal Gyrus – **Right Insula**			-.38	.011					**X**	
	Right Medial Frontal Gyrus – Rostral ACC			-.31	.046						X
	Right Frontal Orbitalis – **Right Insula (OF)**			-.27	.04				X		
	Right Frontal Operculum – **Right Insula (OF)**			-.34	.028					**X**	
	Rostral ACC – Right Frontal Operculum	-.25	.011			X					
	Rostral ACC – **Left Insula**			-.35	.023					**X**	
	Rostral ACC – **Right Insula (OF**)			-.33	.029						X
	Dorsal ACC – Right Frontal Orbitalis	-.20	.049	.22	.03					**X**	
	Dorsal ACC – **Left Insula**	.28	.004							**X**	
	Dorsal ACC – **Right Insula**	-.24	.017				X				
	Dorsal ACC – **Right Insula (OF)**			-.39	.01					**X**	
	**Left Insula** – Right Frontal Operculum	.22	.027							**X**	
	**Left Insula** – Right Frontal Orbitalis	.25	.01							**X**	
	**Left Insula – Right Insula**	.20	.049			X					
	**Left Insula** – **Right Insula**	.24	.017					X			
	**Right Insula** – Right Frontal Operculum	.32	.001					X			
	**Right Insula – Right Insula (OF)**	-.22	.024				X				
Controls	Right Medial Frontal Gyrus – Rostral ACC			-.56	.006	X					
	Right Medial Frontal Gyrus – **Left Insula**	-.34	.026								X
	Right Medial Frontal Gyrus – **Left Insula**			-.58	.012					**X**	

	Right Medial Frontal Gyrus – **Right Insula**			-.57	.013					**X**	
	Right Frontal Operculum – **Right Insula (OF)**			-.48	.045					**X**	
	Rostral ACC – Right Frontal Operculum	-.37	.015	.54	.02	X					X
	Rostral ACC – **Left Insula**	-.37	.015				X				
	Rostral ACC – **Right Insula (OF)**	-.39	.011					X			
	Rostral ACC – **Right Insula**	-.51	<.0001					X			
	Rostral ACC – **Right Insula (OF)**	-.41	.007						X		
	Dorsal ACC – **Right Insula**	-.36	.019				X				
	Dorsal ACC – **Left Insula**	-.33	.03					X			
	Dorsal ACC – **Right Insula (OF)**			.49	.041						X
	**Right Insula** – Right Frontal Operculum	-.31	.043				X				
	**Right Insula** – Right Frontal Orbitalis	-.47	.002				X				
	**Right Insula** – **Right Insula (OF)**	-.35	.021				X				
mTBI	Right Medial Frontal Gyrus – **Right Insula (OF)**	.35	.006				X				
	**Left Insula – Right Insula (OF)**	.26	.044					X			
	**Left Insula – Right Insula**	.31	.016					X			
	Right Medial Frontal Gyrus – Right Frontal Operculum	.27	.041						X		
	Dorsal ACC – Right Frontal Orbitalis	-.26	.046								X
	Rostral ACC – **Left Insula**			-.41	.04					**X**	
	Right Frontal Orbitalis – **Left Insula**			-.57	.003					**X**	
	Right Frontal Orbitalis – **Right Insula (OF)**			-.45	.025						X

Supplementary Table 5. Spearman correlation coefficients for inter-regional functional connectivity and trait (FSS) or state (CE Time Constant) fatigue.

Regional Connection	rho	p	Condition
Rostral ACC – Left Insula	.24	.044	25% 1st Half
Right Insula – Right Insula (OF)	-.29	.015	25% 2nd Half
Left Insula – Right Insula (OF)	-.40	.001	75% 1st Half
Left Insula – Right Insula	-.33	.006	75% 1st Half
Right Medial Frontal Gyrus – Right Insula	-.30	.011	75% 2nd Half
Dorsal ACC – Right Operculum	-.34	.049	75% 2nd Half
Dorsal ACC – Right Insula	-.24	.048	75% 2nd Half

Supplementary Table 6. Correlation between inter-regional functional connectivity and time post onset of injury.

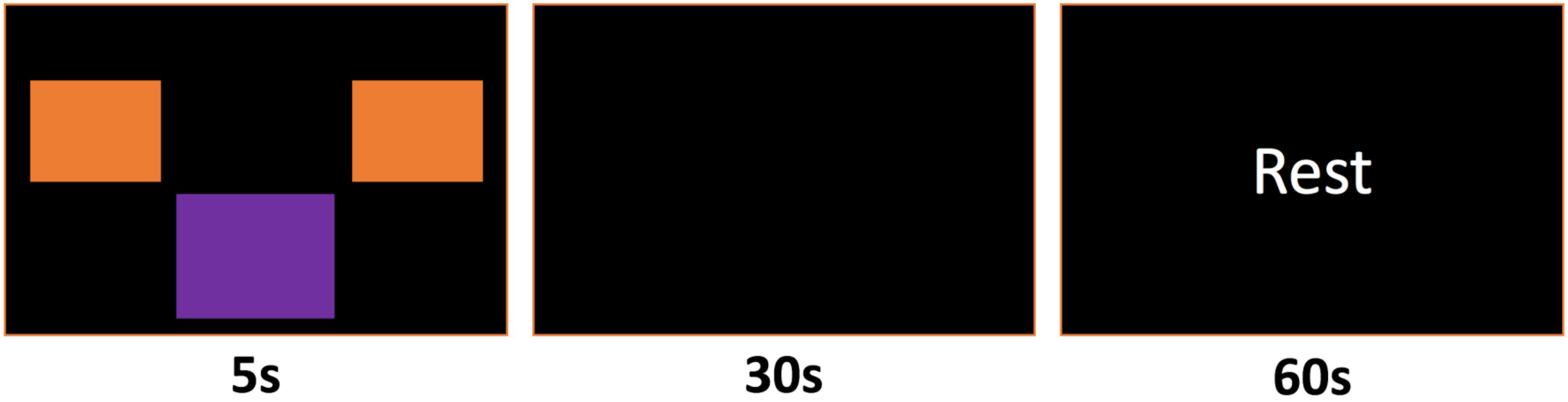

Supplemental Figure 1. In the Constant Effort task, participants squeeze a bulb while viewing a display for 5 seconds to achieve the desired starting level. The display is adjusted such that the middle bar (purple) is moved to match the height of the two stationary bars by adjusting their effort on the bulb. They are then given 5 seconds before a blank screen comes up for 30 seconds during which the subject must maintain the same effort. The participant is then given 60 seconds of rest before the next trial is presented.

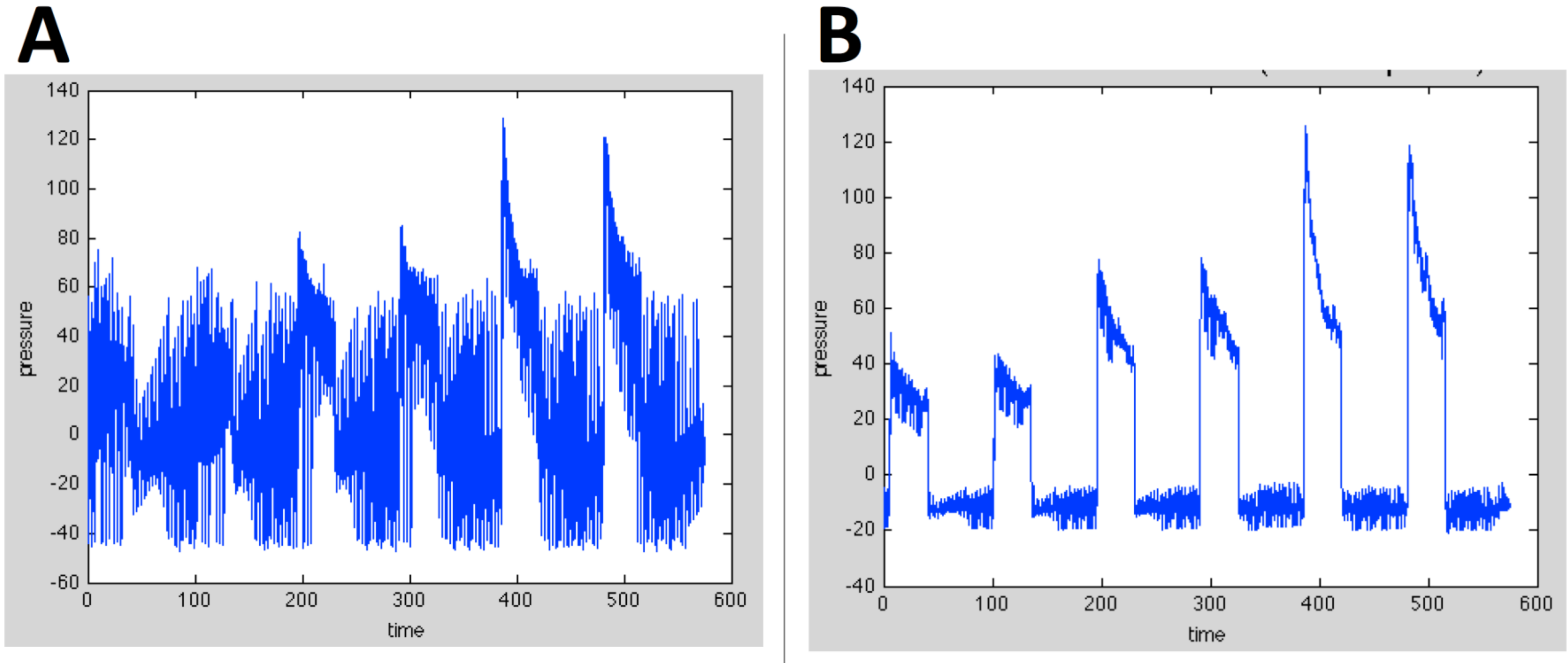

Supplemental Figure 2. The median sliding window filtering algorithm used to smooth the data was used to filter the raw data (A) to an appropriate level (B) for analysis.

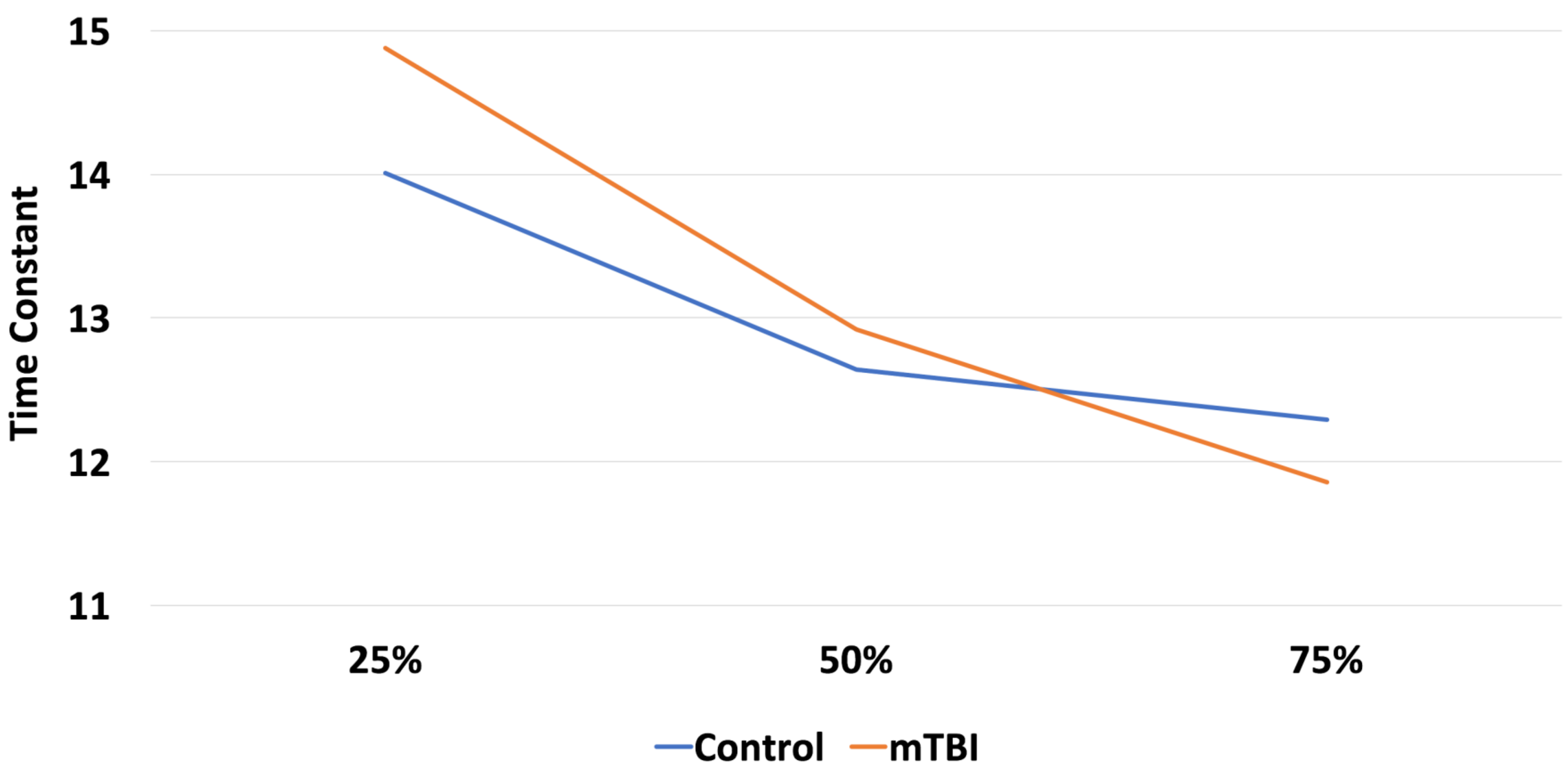

Supplemental Figure 3. Performance on the Constant Effort task was equivalent across groups, but demonstrates an effect of effort level.
